# Ultra-Endurance Participation and Acute Kidney Injury: A Narrative Review

**DOI:** 10.3390/ijerph192416887

**Published:** 2022-12-15

**Authors:** Victoria Tidmas, Jon Brazier, Lindsay Bottoms, Daniel Muniz, Terun Desai, Janine Hawkins, Sivakumar Sridharan, Ken Farrington

**Affiliations:** 1 Department of Psychology, Sport, and Geography, De Havilland Campus, University of Hertfordshire, Hatfield AL10 9EU, UK; 2 Centre for Health Services and Clinical Research, De Havilland Campus, University of Hertfordshire, Hatfield AL10 9EU, UK; 3 Renal Unit, Lister Hospital, East and North Herts Trust, Stevenage SG1 4AB, UK

**Keywords:** ultra-endurance, acute kidney injury, non-steroidal anti-inflammatory drugs, hydration, exertional rhabdomyolysis, exercise-associated hyponatremia

## Abstract

Increasingly popular, ultra-endurance participation exposes athletes to extremely high levels of functional and structural damage. Ultra-endurance athletes commonly develop acute kidney injury (AKI) and other pathologies harmful to kidney health. There is strong evidence that non-steroidal anti-inflammatory drugs, common amongst ultra-athletes, is linked to increased risk and severity of AKI and potentially ischaemic renal injury, i.e., acute tubular necrosis. Ultra-endurance participation also increases the risk of exertional rhabdomyolysis, exercise-associated hyponatremia, and gastrointestinal symptoms, interlinked pathologies all with potential to increase the risk of AKI. Hydration and fuelling both also play a role with the development of multiple pathologies and ultimately AKI, highlighting the need for individualised nutritional and hydration plans to promote athlete health. Faster athletes, supplementing nitrates, and being female also increase the risk of developing AKI in this setting. Serum creatinine criteria do not provide the best indicator for AKI for ultra-athletes therefore further investigations are needed to assess the practicality and accuracy of new renal biomarkers such as neutrophil gelatinase-associated lipocalin (NGAL). The potential of recurring episodes of AKI provide need for further research to assess the longitudinal renal health impact of ultra-participation to provide appropriate advice to athletes, coaches, medical staff, and event organisers.

## 1. Introduction

Since the turn of the millennium ultra-endurance events, defined by distance (>42.195 km for running), duration (>6 h or multiple days/stages), and environment (mountain, desert) [1,2] have increased in popularity [3,4]. As regular exercise including endurance and/or resistance is recommended to promote health benefits and prolong life [5], ultra-endurance athletes are considered to be healthy due to their increased cardiovascular fitness, commitment to high-volume training, and increased energy expenditure [6]. Preliminary evidence suggests repeated extremely strenuous exercise can promote increased mortality and reductions in health benefits in relation to increasing training dose endorsing a J- or U- shaped relationship [7,8]. Of interest to this review is the common development of acute kidney injury (AKI) post-ultra-endurance participation [1,4,7,9]. Although a consensus has not been reached regarding the true incidence of AKI for these athletes due to the multiple and inconsistent methods used [1,3,7].

Ultra-athletes are at increased risk of AKI, especially those participating in high volume and intensity ultra-running events, are exposed to high levels of functional and structural damage [2,4,7], increased serum creatinine (SCr) levels [8] and increasing renal filtration requirements [10]. The ingestion of non-steroidal anti-inflammatory drugs (NSAIDs), which is common in sports medicine [11,12,13] with up to 75% of ultra-athletes reporting NSAIDs use [14,15,16,17] is also a concern. NSAIDs are a contributing factor to ultra-endurance event completion success [16] as they assist in mitigating muscle inflammation, postpone fatigue, and improve pain tolerances [18,19,20]. High doses, equalling the maximal over-the-counter doses, can compromise training adaptations, reduce performance [12,21], increase the risk of AKI and accelerate progression of AKI to chronic kidney disease (CKD) [22]. Additionally, being of female sex, dehydration status [23,24], experiencing significant weight loss during races [25,26] and running faster over shorter durations [14,25] are associated with increased AKI risk for ultra-athletes.

Ultra-endurance events require prolonged extremely strenuous exercise while self-managing nutritional and hydrational status to facilitate optimal physical and mental performance [27]. Sub-optimal nutritional status in ultra-athletes is common and often requires participant withdrawal and/or medical intervention [27]. Conventionally, ultra-athletes do not prioritise protein [28] where nutrition and fuelling mainly focuses on carbohydrates [28,29]. In times of limited energy availability or insufficient protein ultra-events can incite muscle breakdown via inducing a catabolic state [30,31,32], which although uncommon can result in exertional rhabdomyolysis (ER) [4] which can induce kidney tubule damage and AKI [4,33]. Over-hydrating and intolerable fuelling regimes can result in exercise-associated hyponatremia (EAH) [34,35], gastro-intestinal symptoms (GIS) and endotoxemia [36,37] all of which can lead to kidney tubule damage and impaired kidney function.

**Figure 1:**
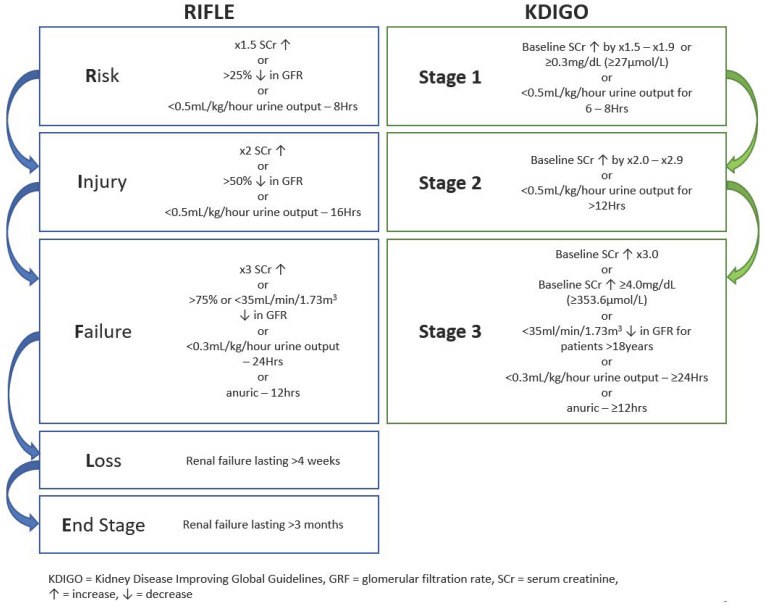
Outline of 2 different criteria for acute kidney injury (AKI); Risk, Injury, Failure, Loss, End-stage (RIFLE) classification (**left**) and the clinical practice guidelines published by Kidney Disease Improving Global Guidelines (KDIGO) for acute kidney injury (**right**).

AKI can be described as a sudden decrease in renal function which if prolonged can result in structural damage to renal tissues and impairment [38] but is mostly asymptomatic and reversible [39]. Increased blood levels of nitrogen waste products are characteristic of AKI [8] and changes SCr, blood urea, and cystatin-C (Cyst-C) levels are indicators of kidney function [40,41]. More novel and less used biomarkers reflecting renal tissue injury are neutrophil gelatinase-associated lipocalin (NGAL) and kidney injury molecule 1 (KIM-1) [33]. However, clinical AKI criteria, such as Risk, Injury, Failure, Loss, End-stage (RIFLE) and Kidney Disease Improving Global Guidelines (KDIGO) for AKI, are founded on changes observed to SCr levels and urine volume (Figure 1) [41,42]. Other structural and subclinical measures have been suggested but their use lacks consensus [43,44] while no established range of values exists for sport participation [4].

## 2. Materials and Methods

An online literature search was conducted utilising three online electronic databases, Google Scholar, PubMed, and ScienceDirect. The design of this review was narrative however, the search strategy and parameters closely followed the PRISMA statement guidelines [45]. The key search terms included, ‘kidney injury’, ‘acute kidney injury’, ‘kidney dysfunction’, ‘impaired kidney function’, and ‘ultra-endurance’, combined to produce search phrases such as, ‘acute kidney injury and ultra-endurance’. To promote a current and relevant review, papers were not included if published prior to 2000, non-peer-reviewed, or written in a language other than English. Those that were reviewed met the following inclusion criteria; ultra-endurance athlete population and included estimates or measures of renal function. Relevant articles published prior to 2000 found in reference lists of reviewed articles were also included or discussed throughout this review.

This search identified 22 articles, briefly outlined in Table 1 (full table of review, Table S1, Supplementary Material), 17 were prospective observational cohort studies [23,25,26,39,40,43,46,47,48,49,50,51,52,53,54,55,56], the remainder comprised of 3 retrospective cases series [34,35,57], and 2 individual case reports [3,58]. This resulted in 445 ultra-athletes meeting the at-risk AKI criteria and 281 meeting the injury AKI criteria. One case of acute tubular necrosis (ATN) was reported. Other conditions were reported alongside renal dysfunction including 43 cases of exertional rhabdomyolysis (ER) in six studies, 30 occurred alongside AKI diagnosis. 21 cases of exercise-associated hyponatremia (EAH) were reported in three studies, 7 occurred alongside AKI diagnosis while another 8 occurred concurrently with both ER and AKI.

## 3. Acute Kidney Injury and Ultra-Endurance Events

Acute kidney injury (AKI) in ultra-endurance events has been frequently reported [1], however, the prevalence of AKI in ultra-endurance athletes has not reached a consensus ranging from 0% [47,59] up to 85% [26]. Differing and inconsistent methodologies relying on changes to serum creatinine (SCr) levels, in addition to observing different modalities of ultra-events for measuring the impact on kidney function during ultra-endurance likely explains variances [3,56]. Recently 96.2% of AKI cases that also occurred in conjunction with exertional rhabdomyolysis (ER) were linked to ultra-running events [2]. The majority of studies in this review (Table 1) pertained to ultra-running events however 3 studies included ultra-cycling participants [53,54,56]. The serum creatinine (SCr) levels for all the participants observed by Chlibkova et al. (2015) in both ultra-mountain bike and ultra-running races increased, however there was a significant increase in post-race SCr for 24 h ultra-running athletes compared to those competing in 24 h mountain biking events (*p* < 0.01). It should be noted that very few ultra-athletes seek or need medical intervention in the presence of an AKI diagnosis [56,60].

**Figure 2:**
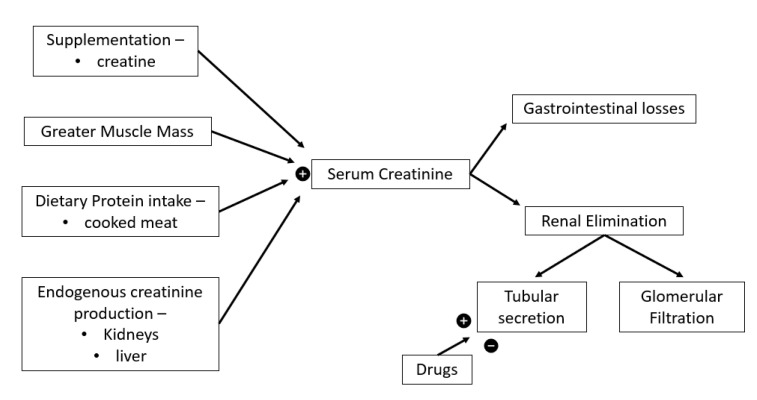
Serum creatinine determinants.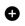 = increase, 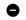 = decrease.

Multiple factors effect SCr levels as seen in Figure 2. Endogenous creatine production occurs within the liver and kidneys (Figure 2) from glycine, methionine, and arginine which is subsequently transported to skeletal, cardiac muscles and other tissues [61] where the non-enzymatic anhydration of creatine results in creatinine [62,63]. High protein diets including cooked meats provide an additional creatine source (Figure 2). Renal elimination of serum creatine occurs via glomerular filtration and low, yet unpredictable levels of tubular secretion, which is affected by multiple drugs, while small gastrointestinal losses also occur [62,63] (Figure 2).

Creatinine clearance is a useful indicator of GFR though overestimates it by around 10% compared the gold standard inulin clearance [62]. Measuring creatinine clearance requires prolonged and impractical urine collection in addition to blood sampling [62], in clinical practice GFR is usually estimated using equations based on SCr modified by sex, age and ethnicity to provide some degree of correction for differences in creatinine generation [64]. SCr GFR equations were generated using the general population or hospitalised patients [65,66] and assume creatinine generation is steady state, not always true (Figure 2), especially during or after extreme exercise [62,64], as significant increases in SCr levels are common post ultra-events (Table 1). Severe muscle damage is common during ultra-endurance events [4] therefore increases in SCr levels can reflect muscle damage rather than reductions in GFR [8,52]. Using SCr as an indicator of renal function produces inaccurate reflections of the impact of exercise on kidney function [67] and may lead to overdiagnosis of AKI in ultra-athletes. Poussel et al. (2020) found using creatinine produced an overestimation of AKI prevalence compared to use the cystatin C. Additionally, the normal GFR for ultra-endurance athletes is unknown due to a lack of baseline data [68] and back-calculating estimates of baseline SCr for ultra-athletes has been shown to be inaccurate [43,68]. Hence, avoiding SCr in settings of extreme exercise has been recommended [8]. This highlights the importance of pre-race testing, participant screening, and the need for a more accurate and valid method for monitoring renal function for this population.

More accurate biomarkers of renal dysfunction are required which can identify abnormal kidney function and discriminate between intrinsic renal disease and prerenal azotaemia which is often induced by strenuous exercise [8]. As there are 3 groups of AKI biomarkers: indicators of renal function (low molecular weight proteins such as Cystatin C) [69], indicators of tubular damage (urinary neutrophil gelatinase-associated lipocalin (NGAL) and urinary kidney injury molecule-1 (KIM-1)) [8] and renal inflammatory mediators (leukocytes and prostaglandins) [70]. Cystatin C (Cyst-C) has less non-GFR determinants, being unrelated to muscle mass or diet, compared to SCr [69,71,72] and has been recommended in populations with increased muscle mass or extreme diets [73]. Over the marathon distance, the rise in Cyst-C was equal to half that of the increase in SCr, supporting that Cyst-C is less affected by sport related renal blood flow changes or muscle damage [74]. Mccullough et al., (2011) found that there was a comparable rise and normalisation for both SCr and Cyst-C after marathon running. Additionally, this study found a 5-fold increase in NGAL and a lesser increase in KIM-1 measures supporting a real decrease in renal function due to renal tissue injury [33]. As an 25 kDa acute phase protein, NGAL is mainly produced in the kidney tubules [8,47,48], is a key indicator of early ischemic damage to the kidneys [75,76,77,78] and early detection of AKI and predicting renal disease progression [48,59,75,76]. NGAL levels have been shown to significantly increase during then returned to baseline within 24 h [8] or continued to increase post ultra-race [33] indicating that tubular injury was present independent of changes to SCr. The minimal increase in urinary neutrophil gelatinase-associated lipocalin (NGAL) and urinary kidney injury molecule-1 (KIM-1) levels in another study also argues against the diagnosis of AKI using criteria based on increased SCr [47]. Therefore, future studies should compare the sensitivity of available biomarkers while also meetings the practical needs of medical professionals present at ultra-endurance events.

## 4. Non-Steroidal Anti-Inflammatory Drugs

**Figure 3:**
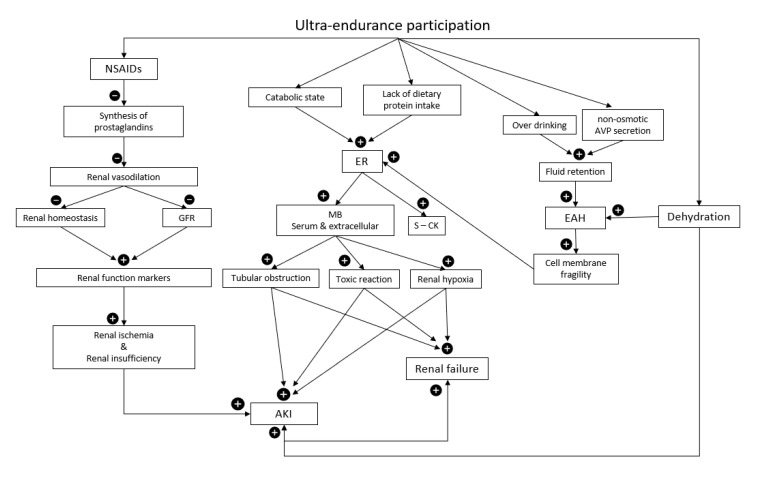
Multiple pathologies and mechanisms promoting acute kidney injury (AKI) in ultra-endurance athletes. Non-steroidal anti-inflammortory drugs (NSAIDs), glomerular filtration rate (GFR), exertional rhabdomyolysis (ER), myoglobin (MB), serum creatine kinase (S-CK), arginine vasopressin (AVP), exercise-associated hyponatremia (EAH), 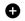 = increase, 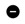 = decrease.

Five studies reported AKI alongside positive use of NSAIDs for ultra-athletes (Table 1). In only one study was use of NSAIDs denied by ultra-athletes [46], whilst the remaining 16 studies did not specify whether there was use of NSAIDs by their participants. NSAIDs inhibits cyclo-oxygenase production thus preventing the synthesis of prostaglandins resulting in increased renal vasodilation and decreasing homeostasis [25,77] (Figure 3). Exercise reduces renal blood flow, however renal perfusion is augmented, and homeostasis maintained via vasodilatory prostaglandins [78]. The absence of prostaglandins and the subsequent reduced renal perfusion results in reduced GFR contributing to mild increases in renal function markers and ultimately, occasionally, kidney failure [67,79]. For healthy individuals, prostaglandin stimulated vasodilation is unnecessary for maintenance of renal function [78], while those with renal disease requires prostaglandin driven vasodilation of renal vascular beds to maintain renal perfusion [80] and avoid renal ischemic injury [78].

Compared to marathon (42 km) and trail runners (67 km), ultramarathon runners (112 km) had the highest per-event NSAIDs intake indicating that the duration of the event plays a role in the quantity and chronological consumption of NSAIDS in ultra-events [17]. Although NSAIDs have been found to be a significant risk factor for AKI after endurance events, some debate exists regarding whether NSAIDs induce a reduced renal function in ultra-athletes. Wharam et al., (2006) found that NSAIDs significantly increase SCr compared to non-NSAIDs users. More recently, Lipman et al., (2014) showed a 18% increase in overall AKI incidence and severity for NSAIDs users, as 12% more ultra-athletes met the AKI at-risk criteria and 6% more met the injury criteria (Figure 1) [14]. Poussel and colleagues found that observing only non-NSAIDs user ultra-athletes reduced the incidence of AKI compared to other ultra-athlete studies [59]. However, contrary evidence exists as both Dumke et al. (2007) and Page et al. (2007) found no significant difference in SCr levels between NSAID using and non-using ultra-runners, though both sample sizes were small, the NSAIDs doses subtherapeutic, and the studies were unblinded.

In the 11 case reports systematically reviewed by Hodgson et al. (2017) investigating the concern of severe AKI after various marathon and ultramarathon events, 22 of 27 cases of severe AKI in ultra-athletes required hospitalisation, 18 (67%) reported NSAIDs use. In the studies discussed in this review (Table 1) no renal biopsies were completed, although 1 case of acute tubular necrosis (ATN) was made from urinalysis, serology, and ultrasound results [58]. However, in studies reviewed by Hodgson et al., (2015), 4 kidney biopsies were completed, all revealing ATN for both marathon and ultramarathon distance events, suggesting an ischaemic aetiological link in developing AKI and ATN for endurance athletes using NSAIDs [8]. NSAIDs have also been linked to the development of EAH and ER in ultra-athletes which may also contribute to subsequent AKI and hospitalisation [34,60]. Ultra-athletes should approach the consumption of NSAIDs with caution and pay closer attention the potential negative health outcome, especially the development of AKI and ATN rather than performance benefits.

## 5. Hydration

Ultra-endurance events occur across the world and expose athletes to a multitude of different and fluctuating environmental conditions including hot, humid, tropical conditions [4,26,34,35,81]. Physical exertion in these ambient conditions often promotes kidney damage and ultra-athletes often experience inadequate hydration status [39,81,82]. Altered kidney function is promoted due to combinations of dehydration, high internal and external metabolic loads, and heat strain [24,83]. Severe dehydration is widely recognized as a contributor to AKI after endurance exercise and other life-threatening consequences [23,24]. Increased loss of electrolytes and water via increased sweating can also cause hyponatremia [34,56]. While adequate hydration status maintenance does not prevent ER in ultra-athletes [26], dehydration induced reduction of renal blood flow and renal ischemia resulting in an accumulation of muscle proteins within a reduced total blood volume can increase the risk AKI [84,85] and ER [86,87] for ultra-athletes (Figure 3).

Loss of body mass does not directly translate into an absolute measure of dehydration however it is a simple method of assessing hydration status during exercise [88]. Previous studies have found that limiting the body exercise induced weight loss to ~2–3% is necessary to avoid dehydration while maintaining or improving performance [89,90,91]. The average body weight loss for ultra-marathon runners was ~5%, reaching >11% of body weight in some cases [26,92]. Water can be produced during exercise via a combination of the utilisation of endogenous and exogenous substrates and the release of glycogen bound water through glycogenolysis [91] assisting in alleviating dehydration [93]. Therefore, maintaining euhydration requires at least ~2–5% reduction in body mass during ultra-events [91], which seems to be tolerated by ultra-athletes [94]. Belli et al., (2018) found that athletes who presented with body mass reductions >5% (−5.2 to −10.4%) also presented with >25% (−27.1 to −44.4%) reduced GFR from 84 km to the completion of the 200 km event. Similarly, a greater reduction in body mass from 120 km was linked to greater renal dysfunction for ultra-athletes during multi-stage 250 km desert races [25]. Hence, complying with typical hydration guidelines and avoiding a 2% mass loss [30,95], can result in hyperhydration of ultra-athletes [82], increases the risk of developing symptomatic EAH [96], and increased levels of cytokinemia and bacterial endotoxemia and subsequent increased AKI risk [97]. Dehydration is most likely to occur during the early stages of ultra-events as the first 4 h showed the maximum slope of weight loss [25,26,46]. However, an increased total percentage body mass loss resulted in a higher proportion of subjects meeting RIFLE criteria for AKI compared to those with less mass loss [39,57] and therefore needs further investigation.

## 6. Exertional Rhabdomyolysis

Exertional rhabdomyolysis (ER) is a pathophysiological condition described by damage to or necrosis of the striated muscle tissues during strenuous exercise leading muscle cell disintegration enabling the release of myoglobin (Mb) into the bloodstream and extracellular space [98]. Additionally, sarcoplasmic proteins such as S-CK, serum lactate dehydrogenase (S-LDH), aspartate transaminase (AST) and electrolytes are released [99]. In extreme cases of muscle necrosis, ER symptoms can present as weakness, oedema, myalgia, and reddish-brown or tea coloured urine, without haematuria [34]. Six papers were found discussing exertional rhabdomyolysis in ultra-endurance events resulting in 43 cases (Table 1). Of these, 30 cases of AKI occurred either alongside or potentially due to exertional rhabdomyolysis (ER).

Serum Mb can potentially result in kidney failure via 3 mechanisms: tubular obstruction, toxic reaction, and decreased oxygen supply due to vasoconstriction of renal tissues [60,87] (Figure 3). Mb is quickly eliminated from the bloodstream, so S-CK is the preferred marker for ER identification [23] which shows a peak 24–36 h post-exercise with recovery to baseline values at 48–72 h post-exercise [100,101,102]. In ER, Mb and S-CK, can rise ≥4–5 times and sometimes much greater, above normal values, while S-LDH and AST generally only double [103]. A recent systematic review recommended that S-CK > 1000 UI/L as an essential criterion for the diagnosis of ER diagnosis [104].

With an incidence of ~29.9 per 100,000 patient years, ER is relatively uncommon [105] however the concurrent complication of AKI which is significantly more common is a concern for ultra-athletes [86]. Hodgson et al. (2017) reported 27 cases of AKI across varying endurance disciplines (six in ultra-endurance athletes), with concurrent rhabdomyolysis in 23 cases (85%). Not surprisingly ER can lead to several other serious conditions, hyperkalaemia, hypernatremia, acidosis kidney tubule damage and the development of acute and chronic renal injuries [4]. ER due to heat stress and AKI has been reported, indicating that dehydration, hyperthermia, and eccentric muscle loads can promote and contribute to the development of ER + AKI [23,39]. As ultra-endurance events are often held in areas with hot and humid conditions for example the tropics, these conditions can increase the risk of ER [26,34,87]. It has been shown that increasingly prolonged exercise under these thermally challenging conditions can induce increased severity of both ER and AKI [4].

## 7. Exercise-Associated Hyponatremia

Exercise-associated hyponatremia (EAH) was initially reported in ultra-endurance athletes and is a potentially serious condition [106,107]. Symptomatic EAH is caused by fluid overload from continued over-drinking [77,108]. Previous and outdated recommendations to drink as much as possible during exercise [109] resulted in increased occurrences of EAH within athletes [110,111,112]. Blood testing is needed to identify asymptomatic EAH [77], however EAH can present with symptoms such as, vomiting, dyspnoea, changes to consciousness, and convulsions related to hyponatraemic encephalopathy [112]. Hyponatraemic death has been reported post ultra-endurance events [77,107]. Three studies were found to discuss EAH in the presence of AKI for ultra-endurance athletes. A total of 21 cases of EAH were found, 7 of which occurred alongside AKI while 8 cases occurred concurrently with both AKI and ER (Table 1).

Asymptomatic and symptomatic EAH is identified by a low blood sodium concentration (Na^+^ < 135 mmol/L) during or immediately post exercise [107]. Severe hyponatraemia produces water influx into the cells leading to cellular swelling, muscle cell membranes destabilisation, and ultimately traumatic cell rupture during exercise [35,113]. Increased muscle cell fragility can promote the breakdown of muscle tissues, ER, and the subsequent AKI [35,113] (Figure 3). Muscle cell lysis due to strenuous exercise [113,114] or increased thermal strain [115] can result in the third spacing of fluids which in turn can stimulate the secretion of arginine vasopressin (AVP) which facilitates the development of EAH [34]. AVP can be stimulated by pain, emotion, exercise, nausea, heat stress, hypoglycaemia, medications (NSAIDs) [107,116,117], and elevated inflammatory cytokines all of which have been found in ultra-athletes [118]. Over-drinking beyond the feelings of thirst in conjunction with non-osmotic AVP secretion induced by protracted endurance exercise results in fluid retention and EAH [119].

The impact of increased sodium loss via increased sweating on the development of EAH is still controversial [107]. Endurance athletes compared to the general population have reduced levels of sweat sodium, however sweat sodium loss is extremely variable for individuals [120]. An increase in serum sodium would be expected through loss of hypotonic sweat in opposition to EAH, although sweat loss may lead to EAH via fluid loss induced volume depletion stimulating AVP and increased water retention or the ingestion of greatly more hypotonic fluids compared to fluid loss, potentially promoting a net weight loss [107,121]. The link between EAH and ER may be casual or independent [34,103]. Renal function fluctuations can also play a part in the development of EAH, as prolonged oliguria and anuria can result in dilutional hyponatremia and further increase renal compromise [122]. It has been reported that hyponatraemic athletes are more likely to develop ER compared to normonatraemic athletes [123]. ER and EAH may be aetiological factors for the development of either condition [107]. However, each condition requires opposing treatments [34,107], mild EAH requires water restriction protocols while severe EAH need intravenous concentrated bolus of hypertonic saline [107]. ER requires aggressive intravenous isotonic fluids to limit the myoglobin build-up within the renal tissues and AKI [113]. Highlighting the importance of individualised hydration strategies to alleviate risk of EAH and increased awareness of the different treatments needed for opposing conditions.

## 8. Gastrointestinal Dysfunction

Ultra-endurance events require prolonged extremely strenuous exercise while also self-managing nutritional and hydrational status to facilitate optimal physical and mental performance [27]. Sub-optimal nutritional status in ultra-athletes is common and often requires participant withdrawal and/or medical intervention [27]. Gastrointestinal symptoms (GIS) are common in ultra-endurance events with an incidence of 60–93% for both severe upper and lower GIS plus acute abdominal pain, dizziness, and nausea [124,125,126,127]. GIS are linked with hindered capability to meet nutritional and energy demands, poor performance and withdrawal [16,124]. This highlights the need for effective fuelling, hydration, and nutritional strategies for optimal ultra- performance and athlete safety [27]. Ingestion of carbohydrate (CHO) during ultra-endurance events improves performance [128,129]. Indeed, ultra-athlete’s nutrition and fuelling strategies during events mainly focuses on carbohydrates (CHO) [28]. CHO account for ≥70% total daily energy intake equating to >10 gCHO/kg/day [130,131]. However, during extended strenuous exercise, to avoid malabsorption and GIS, fuelling needs to account for the total CHO oxidative capabilities of muscles in the context of individual gastrointestinal tolerance [132].

Blood redistribution away from central organs (gut, liver, and kidneys) to active muscles (oxygen and substrate supply) and skin (heat dissipation) occurs during extended strenuous exercise [31,125]. Depending on exercise intensity, the renal blood flow can be reduced to ~25% of resting values while GFR is maintained [133] and splanchnic blood flow is reduced to ~20% of resting values [134]. Severe gut under perfusion can cause shock-induced mucosal damage, releasing Gram-negative intestinal bacteria and/or endotoxins like lipo-polysaccharides (LPS) which can leak into central circulation if portal clearance is overwhelmed [36,37,135] resulting in endotoxemia [15,31,125,135]. Impaired renal function is commonly reported due endotoxemia [136]. Symptoms such as fever, shivering, dizziness, nausea may occur along with GIS including vomiting and diarrhoea, and finally sepsis [31,135,137]. In vivo, LPS are key activation molecules for the host immune response by inciting the cytokine network during exercise [15,125]. Endotoxins can enter the circulation post-ultra-endurance participation via increased gastrointestinal permeability resulting from splanchnic ischemia, which worsens in the presence of hyperthermia and muscle damage [15,31,125]. Intense exercise can result in immune suppression allowing increasing LPS concentrations exceeding systemic inflammatory response thresholds [138]. Ultimately, disseminated intravascular coagulation, multi-organ failure, and central nervous system disturbances may occur in heat stroke victims [37]. Significantly, endotoxemia has been reported mainly when intense exercise was performed in warm not cool conditions [37,139]. Individual exercise-induced increases in cytokine levels vary considerably and are further increased by NSAIDs consumption [15]. Physiological responses to endotoxins require LPS binding proteins, which can induce a pro-inflammatory response increasing tubular apoptosis and renal function impairment [36,37]. Avoiding GIS via establishing individual gastrointestinal tolerances and fuelling strategies could reduce the risk of developing sub-nutritional status and potentially reduce the risk of AKI for ultra-athletes.

## 9. Beetroot/Nitrates

Medically, dietary nitrates are used to treat conditions such as hypertension and cardiovascular disease [140]. The findings of a pivotal study [141] showing that increased dietary nitrates was associated with a decrease in oxygen cost during submaximal exercise. This significantly increased the popularity of nitrates in endurance athletes [28]. Beetroots and beetroot juice, exemplify several vegetables that provide high levels of inorganic nitrate (NO3^-^) [28] which is ultimately converted to nitric oxide (NO) within the gut [142,143]. Numerous body systems important for endurance exercise performance are affected by NO consumption for example, oxygen regulation in working muscles, blood flow, vasodilation, mitochondrial respiration and biogenesis, overall muscle contractions and relaxation, and glucose uptake [140,142,144]. Cumulatively, this results in improved endurance performance [145].

Nitrate clearance within humans is relatively unknown however it does pass easily through the glomerulus and is mostly reabsorbed at the proximal tubules [143]. Ingestion of 3.5 mmol nitrate was completely removed within 48 h in humans, of which 60% was excreted via the urine [146]. The rate of excretion of urinary nitrates has been used to monitor endogenous NO synthesis in several diseases [147,148,149]. Although this assumes that altered renal function has no effect on renal nitrate excretion or other non-renal pathways, although evidence suggests that fractional excretion of nitrates falls as renal function declines [143]. NO is a gaseous signalling molecule and conditional on its concentration, release location and period of action, has several functions within the kidney [150]. Reductions or absence of a specific isoform of NO synthase production can indicate renal endothelial damage post ischemia-reperfusion [150,151], which is the most common cause of acute tubular necrosis AKI [149].

Although nitrates are fairly non-toxic, high doses of their metabolites (e.g., nitrite, nitric oxide, and N-nitroso compounds) pose an increased concern over potentially promoting carcinogenesis [152]. An increased risk of kidney cell carcinoma has been reported when exposed to excessive amounts of nitrate and nitric oxide [153,154,155]. Dietary nitrate converted into nitrite has the potential to interreact with dietary amines, forming carcinogenic nitrosamines [156]. Nitrate supplementation during resting and submaximal endurance elicited normal kidney function responses to exercise compared to placebo [157]. Post-exercise recovery (60 min) was slightly reduced in the nitrate supplementation group, where the glomerular membrane permeability remained increased compared to the placebo, no longer term effect was reported [157]. It remains those ultra-athletes should be cautious when considering or using uncontrolled quantities of nitrate supplements [156].

## 10. Females

Female athletes are often excluded in studies due to menstrual cycle phases which may confuse physiological results [1]. Female gender is underrepresented compared to males in studies in Table 1 though this is a known risk factor for developing AKI in ultra-athletes [25]. Female athletes are up to 4 times more likely to develop AKI than males, which is similar to and may in part be due to the increased risk of developing EAH in females [158,159]. Bruso et al. (2010) speculated that the lack of women reported who developed EAH and rhabdomyolysis and the subsequent AKI in this paper was due to the numbers of female finishers, ratio (1:5) of females to males, respectively. Nevertheless, EAH and ER have been reported in women [160]. The true mechanisms for this increased risk and association of EAH and AKI in females needs further study [25]. A study including ~50% female participants, suggested that females were unable to rehydrate during endurance exercise as well as males [161].

Female ultra-athletes also struggle meeting the increased daily caloric requirements of ultra-training and competition due to the interaction of sex-hormones and energy availability [1]. High training volumes and/or periods of calorie intake restriction can occur in ultra-athletes causing impairment of physiological functions, known as the syndrome of relative energy deficiency in sport (RED-S) [162]. Both male and female athletes can experience the detrimental effects of a low energy availability, however female athletes feel the effects more rapidly and to a greater extent due to the Female Triad a relationship between menstrual cycle fluctuations and bone health [163]. Additionally, the high training mileage and low bone density associated with oestrogen deficiency can increase the risk of bone fractures for females [1]. The incorporation of females and the increased risk of AKI may potentially skew results or overestimate the true prevalence/risk of AKI for the whole ultra-endurance population, in which males make up the majority of participants [77].

## 11. Hormones

Ultra-endurance participation promotes stress indicated by changes in the hypothalamic-pituitary-testicular (HPT) axis shown by decreased testosterone, luteinizing hormone, and sex hormone-binding globulin levels, while increased cortisol levels and reductions in testosterone: cortisol ratio indicates a catabolic state [84,98,164]. Ultra-marathon running activates both the coagulation and fibrinolytic systems in male and female runners which significantly increases stress on kidney function [165].

Increased cortisol levels immediately post ultra-endurance events is a key marker of stress experienced by athletes [84,98,166]. Kupchak et al. (2014) showed greater cortisol concentrations increase (4.3-fold) after a 161 km trail run than in previous reports over marathon running [167,168], Ironman triathlon [166], and ultramarathon finishers [169]. Multiple factors could be the cause for this greater increase in cortisol such as the increased duration [170], quantity of time in eccentric downhill running and changes in altitude [46], variable temperature conditions [170,171] and experience of energy deficiency [172]. The intensity of ultra-marathon performance has also been linked to higher cortisol levels corresponding with athletes with higher running speeds [173]. Cortisol provides an immunosuppressive effect, assists in increasing blood glucose levels and the metabolism of fats and proteins [98] and increased serum cortisol level is protective against injury [174]. The increased levels of cortisol could provide a protective factor against renal injury during ultra-endurance events.

## 12. Ultra-Endurance Performance

Within non-athletes, the occurrence of AKI is an indicator of increased susceptibility to future AKI events and the development of CKD [57]. Mild AKI insults are common in ultra-endurance athletes, therefore the suggestion that recurring participation could result in an increased risk of developing future AKI and/or increasing the severity of the subsequent insult [57,84]. Although, assessing AKI via clinical criteria suggests that up to 82% of ultra-endurance athletes may be affected, these characteristics tend to resolve within 24 h post finish [6,14,23,33]. Renal chemistries observed post 56 km ultra-marathon showed a full recovery overnight [52], while kidney function often recovers to baseline between stages of multi-stage ultra-events [39]. Experiencing reduced renal function during an ultra-endurance event or due to participation in multiple events currently shows no negative cumulative effects and does not lead to any apparent future renal function restrictions [39,51,57]. However, clinical AKI criteria have been established during resting conditions of clinical populations, utilising this for ultra-endurance populations may lead to an overestimation of AKI and an inappropriate method for ultra-endurance athletes [41].

Ultra-athletes should be aware that shorter and faster ultra-marathons have greater likelihood of causing reductions in kidney function than longer and slower events [14,23,25,40,57,84]. Originally, an increased AKI incidence was reported for marathons runners, up to 84% of participants meeting AKI criteria [6,10,33], where the main differences between marathon and ultra-marathons runners are that the latter run at much slower speeds and have increased training hours and kilometres [85,175]. Within an ultra-endurance cohort, the slowest finishers were found to be less likely to develop AKI [14,25], compared to the top 10 fastest finishers, the AKI risk was decreased up to 82% in slower finishers [25]. A greater percentage of those with increased blood measures indicating AKI were among the faster finishers [57]. Faster finishers are also at increased risk of increased severity of AKI with more athletes meeting stage 2 criteria (Figure 1) than slower finishers [23]. Several factors could contribute to this increased risk, such as when assessing the speed variability during 100 km ultra-marathon suggested that glycogen depletion related fatigue at 40th–50th km when running ~65% VO^2^max may play a part in increasing AKI risk [40]. Additionally, the longer exposure time of slower athletes provides more hydration opportunities potentially attenuating the risk of renal injury due to dehydration [25]. Lower myoglobin levels have also been reported for slower finishers, which suggests a reduced renal damage due to myoglobinemia [176], however this was not reflected in a previous study [177]. Ultra-athletes should consider the health risk of competing in shorter, faster paced events, while more research is needed to provide a clearer understanding if repeated participation imparts increased risk of recurrent AKI, increased severity and/or the progression to CKD.

## 13. Future Considerations

Ultra-endurance events expose athletes to various extreme weather conditions due to remote locations and large distances covered by athletes, which hinders the accuracy of athlete health and safety monitoring by event and medical staff [1]. Using modern technologies, GPS tracking, or heart rate monitors can provide continued participant monitoring (training and competitive intensities, volumes, and recovery) and earlier identification of potentially serious adverse events [7]. Mandatory pre-event screening using an agreed, accurate, and appropriate method of measuring renal function for ultra-athletes should be conducted identify those at increased risk of renal dysfunction [8].

The use of new and more accurate biomarkers of renal function, Cyst-C, NGAL and/or KIM-1, can provide athlete teams, event organisers, medical staff, and researchers with increased quality and accuracy of data reflecting real race settings and longitudinal observational opportunities [1,4,8]. However, the remote nature of ultra-events means that practicality of medical assessment tools is vital. Of interest, Hoffman et al. (2013) collected post-race blood and urine samples were collected for 150 ultra-athletes where the urine dipstick tests showed a trace of protein in 76%, 3+ haemolysed blood in 62%, and measurable ketones were found in 39% of results. This study found that urine dipstick results inclusive of at least 1+ protein, 3+ blood, and specific gravity ≤ 1.025 identified participants meeting AKI ‘injury’ criteria with a sensitivity of 1.0 and specificity of 0.76. Blood creatine kinase concentrations in those meeting AKI criteria was higher than those at AKI risk and creatine kinase levels > 20,000 U/L was deemed an acceptable threshold to provide treatment preventing kidney failure due to rhabdomyolysis [60]. These finding suggest that simple dipstick urinalysis may offer a rapid and inexpensive method to evaluate susceptibility for AKI post ultra-endurance event. For medical support this information is key, as ultra-athletes are exposed to various combinations of dehydration, exertional rhabdomyolysis, and exercise-associated hyponatremia (EAH) some of which require rapid potentially opposing treatment protocols [34,107,113].

## 14. Conclusions

There is strong evidence that NSAIDs use is linked with increased risk of AKI and the progression of renal impairment to CKD for the general population, the common practise of NSAIDs consumption for ultra-endurance athletes is concerning and more research is needed to identify the longitudinal impact on ultra-athlete’s kidney health. Ultra-endurance athletes are also at increased risk of developing multiple different and interlinked pathologies such as ER, EAH, and GIS, which all have the potential to increase risk of developing AKI. Hydration and fuelling seem to play a role in the development of multiple pathologies as well as AKI, therefore individualised nutritional and hydration plans are key for athlete health. Further research is required to assess the longitudinal renal health impact of ultra-participation and to identify the most appropriate and accurate biomarkers and method of measuring renal function for ultra-athletes to enable the provision of suitable advice to athletes, coaches, event organisers, and medical staff.

## Figures and Tables

**Table 1:** Overview of studies examining the occurrence of AKI, Exertional rhabdomyolysis, and Exercise-associated Hyponatremia in ultra-endurance athletes.

Study	Cases	Event	Pre–Race	Diagnosis	Follow-up/ Recovery	NSAIDs
35	*n* = 4	Ultramarathon (89.3 km)	NS	4/4 = AKI 4/4 = ER 3/4 = EAH	Hospitalization and acute renal dialysis (2–21 days)	3/4 = YES
34	*n* = 5	Ultramarathon (161 km)	NS	3/5 = AKI 5/5 = ER 5/5 = EAH	Intravenous fluids Hospitalization	4/5 = Yes
23	*n* = 26	Ultramarathon (100 km)	Yes	22/26 = AKI 18/22 = Stage I AKI 4/22 = Stage II AKI 26/26 = ER	NS	Positive history
57	*n* = 627	Ultramarathon (161 km)	Yes	227/627 = AKI risk 31/627 = AKI	NS	NS
39	*n* = 30	Multi-stage Ultramarathon (177 km)	Yes	55–85% = RIFLE criteria Total over 3 check points 31 = AKI risk 6 = AKI	Normalised prior to start of each new stage—24 h	NS
26	*n* = 26	Ultramarathon (100 km)	Yes	22/26 = AKI 18/26 = stage I AKI 4/26 = stage II AKI	Hospitalisation—1 day	NS
46	*n* = 6	Ultramarathon (217 km)	Yes	RIFLE risk only met by reduction to GFR (>25%) from 84 km no change in SCr	No medical intervention needed	Negative use
40	*n* = 50	Marathon & Ultramarathon (100 km & 308 km)	YES	100 km (*n* = 17) = greatest increase in SCr, all above upper reference limit	NS	NS
58	*n* = 1	Ultramarathon (90 km)	NS	Acute tubular necrosis (ATN), ARF, & ER	Hospitalisation—10 days	Yes
47	*n* = 47	Ultramarathon (80 km)	Yes	Total over 3 check points 8 = Stage I AKI 1 = Stage II AKI	Day 9 = normalised	NS
25	*n* = 128	Multi-stage Ultramarathons	Yes	80–102/128 = AKI per check point Total over 3 check points 179 = AKI Risk 101 = AKI	NS	NS
43	*n* = 37	Ultramarathon (100 km)	Yes	18/37 = AKI	NS	Yes
48	*n* = 16	Ultramarathon (60 km)	Yes	6/16 = AKI	NS	NS
49	*n* = 13	Ultramarathon (149 km)	Yes	SCr = increased ~1/3	NS	NS
50	*n* = 2	2-man- relay 24 h ultramarathon	Yes	SCr = increased Cr clearance = reduced	SCr = normal after 24 h	NS
51	*n* = 5	Ultramarathon (90 km)	Yes	1 = female = collapsed with transient oliguria with renal tubular dysfunction	Renal dysfunction persisted = 14 days Full recovery = 1 year	NS
52	*n* = 8	Ultramarathon	Yes	SCr = significantly increased	24 h = returned to baseline	NS
53	*n* = 28	Ultra-cycle	Yes	SCr = significantly increased	SCr elevated at 24 h	NS
54	*n* = 16	Ultra-cycle	Yes	SCr = significantly increased	SCr normalised at 24 h	NS
55	*n* = 10	Ultramarathon	Yes	SCr post-race = no significant increase SCr increase by 25% at 6 h post-race	SCr normalised at 48 h	NS
56	*n* = 113	Ultramarathon & ultra-mountain bike (24 h & 100 km)	Yes	2/113 = increased SCr = AKI 13/113 = EAH 6/113 = ER 2/113 = EAH & ER = runners	No medical intervention	NS
3	*n* = 1	Multi-stage ultra-trail marathon (786 km)	Yes	ER No AKI	9 days	NS

AKI = acute kidney injury, ER = exertional rhabdomyolysis, EAH = exercise-associated hyponatremia, SCr = serum creatinine, NS = not specified.

## Data Availability

Not applicable.

## Supplementary Materials

The following supporting information can be downloaded at: https://www.mdpi.com/article/10.3390/ijerph192416887/s1, Table S1: Overview of studies examining the occurrence of AKI, Exertional rhabdomyolysis, and Exercise-associated Hyponatremia in ultra-endurance athletes.
