# Supplementary material for: Ultra-Endurance Participation and Acute Kidney Injury: A Narrative Review
Source: Int J Environ Res Public Health. 2022 Dec 15;19(24):16887. doi: 10.3390/ijerph192416887 (PMC9779673; doi:10.3390/ijerph192416887)
Supplement: Supplementary file 1 [file ijerph-19-16887-s001.zip › ijerph-1995754-supplementary.pdf]

Supplementary Table S1. Overview of studies examining the occurrence of AKI, Exertional rhabdomyolysis, and Exercise-associated Hyponatremia in ultra-endurance athletes.

Study	Participants	Distance event	Pre-race measures	Measures at presentation	Results	Environmental conditions	Return to baseline/ Renal follow up info	NSAIDS (Y/N)	Supplements
Boulter 2011	Active male (n = 4) (Age: 35 ± 6y)<br>3/4 = novice ultra-runners	89.3 km, endurance running	Not measured	3/4 = delayed presentation to medical staff<br>3/4 = vomiting & muscle soreness<br>Initial blood samples:<br>3/4 = hyponatraemic (blood sodium concentrations <135 mEq/l)<br>2/4 = hypochloraemic (blood chloride concentrations <98 mEq/l)<br>S-CK (5718–54,231 UI/L)<br>S-Cr (2.99– 12.88 mg/dL)	ER+ AKI<br><br>4/4 - Rhabdomyolysis - presented 1-4 days post-race<br><br>3/4 – EAH<br><br>4/4 – AKI	14–24°C, 63% relative humidity	Hospitalization and acute renal dialysis – between 2-21 days	3/4 = NSAIDs during race<br>1/4 = analgesic/muscle relaxant (Besemax; 450 mg paracetamol and 35 mg orphenadrine citrate) during and Voltaren (NSAID) prior to the race for a knee injury	All = anti-cramp electrolyte supplement (sodium, potassium, magnesium and calcium) 6 to 16 tablets, with an average intake of 11 tablets (1 per hour)
Bruo 2010	Active males (n = 5) (Age: 36.7 ± 7.8 y)	161 km, 5500 m cumulative climb, endurance running	Not measured	Muscle soreness<br>Dark urine<br>S-Cr (1.1–4.9 mg/ dL)<br>S-CK (38,218– 95,940 UI/L)	5 cases of EAH<br>5 cases of ER<br>3/5 progressed to AKI- failure	15–37°C	Intravenous fluids<br>Hospitalization	4/5 = positive NSAIDs during race	NS
Hou 2015	Active males (n = 26)	100 km Ultramarathon	BUN (mmol/L) - 15.23 ± 2.89<br>Creatinine (mmol/L)- 0.94 ± 0.11<br>Myoglobin (μg/L)- 48.9 ± 20.63	BUN (mmol/L) - 24.04 ± 5.49<br>Creatinine (mmol/L)- 1.56 ± 0.39<br>Myoglobin (μg/L)- 4462.46 ± 3391.87<br>CK (U/L) - 4274.81 ± 5903.85	26 = ER<br>22/26 = AKI<br>18 =Stage I AKI<br>4 =stage II AKI<br><br>Faster run = increased risk of AKI	Temperature - 24.9°C (7–8 AM) and 28.7°C (5–6 PM<br>relative humidity was between 66% (10–11 AM)	NS	Positive history of NSAIDs but NS	NS

			CK (U/L) - 166.73 ± 96.91			and 87% (8–9 AM			
Hoffman & Weiss 2016	Active runners (n = 627)	161 km, 5500 m cumulative climb, Ultramarathon	NS	S-CK (120–200,000 IU/L)<br>S-Cr (0.4–2.2 mg/ dL)	AKI<br><br>227 (36.2%) = AKI risk<br>31 (4.9%) = AKI	Outlined in previous refs	NS	NS	
Lipman 2014	Active runners (n = 30)	Ultramarathon (7 days-6 stages-177 km)	SCr (mg/dl) - 0.9 ± 0.2<br>GFR (mg/dl) - 102.7 ± 17.3	Stage 1 finish –<br>SCr (mg/dl) - 1.4 ± 0.4<br>GFR (mg/dl) - 76.6 ± 18.3<br>ΔGFR (mg/dl) - 32.5 ± 18.9 (p = 0.002)<br>Stage 3 finish –<br>SCr (mg/dl) - 1.3 ± 0.4<br>GFR (mg/dl) - 74.5 ± 17.2<br>ΔGFR (mg/dl) - 28.1 ± 20 (p = 0.002)<br>Stage 5 finish –<br>SCr (mg/dl) - 1.4 ± 0.3<br>GFR (mg/dl) - 71.8 ± 19.6<br>ΔGFR (mg/dl) - 30.9 ± 17.5 (p < 0.001)	AKI = 55–85% all participants meet RIFLE criteria<br><br>stage 1:<br>AKI risk = 13<br>AKI = 3<br><br>stage 3:<br>AKI risk = 10<br>AKI = 2<br>stage 5:<br>AKI risk = 8<br>AKI = 1	NS	Before the start of each new stage<br>After 24hrs	NS	NS
Kao 2015	Active males (n = 26) (age: 49y [22–60 y])	100k ultramarathon -not strict entry requirements -first ultra-event on 400m running track organised in Taiwan- to increase entries	S- CK (U/L) - 146 (112-207)<br>SCr (mg/dL) - 0.91 (0.87-1.03)	S-CK - 2299 (1497-4650 UI/L)<br>S-Cr - 1.50 (1.25-1.77 mg/dL)<br>17/26 = moderate dehydration<br>6/26 = muscle cramps<br>3/26 = haematuria	AKI = 22/26 subjects (85%) immediately after race<br>4/26 = no AKI<br>18/26 = stage I AKI<br>4/26 (15%) = stage II AKI	temperature ranged from 24.9–28.7°C average humidity between 66% (10-11 AM) and 87% (8-9 AM)	1 day hospitalization	NS	NS

		Best marathon time= <4hrs All except 2 had done 1 ultra-event before							
Belli 2018	Active males (n = 6)	Brazil 135 Ultramarathon (217km)	S-CK (U/L) – 132 ± 18<br>SCr (mg/dL) – 1.00 ± 0.03<br>GFR (ml/min/1.73m^2) – 89 ± 5	At 134km (equivalent flat distance)<br>S-CK (UI/L) – 3988 ±1004<br>S-Cr (mg/ dL) – 1.34 ± 0.1<br>GFR (ml/min/1.73m^2) – 66 ± 8<br><br>At 279Km (equivalent flat distance)<br>S-CK (UI/L) – 18667 ± 10664<br>S-Cr (mg/ dL) – 1.27 ± 0.08<br>GFR (ml/min/1.73m^2) – 69 ± 5<br><br>At 339Km (equivalent flat distance)<br>S-CK (UI/L) – 19157 ± 12369<br>S-Cr (mg/ dL) – 1.33 ± 0.08<br>GFR (ml/min/1.73m^2) - 65 ± 5<br><br>Muscle soreness	AKI - meet the risk criteria for acute kidney injury from 84 km until they finished the race<br>SCr change did not meet AKI criteria<br>GFR change did meet AKI criteria (>25% decrease)	8–30◦C	NS	Negative use of daily medication - NSAIDs NS	NS
Shin 2016	Active male runner (n =50) (Age:	Marathon (n=17),	BUN<br>Marathon - 17.70±4.24	Post<br>BUN<br>Marathon - 22.79±5.58	Indication of reduced renal function	Starting temperature	NS	NS	NS

	51.76 ± 6.88y)	100 km (n=17) and 308 (n=16) km ultramarathon	100 km- 17.34±3.69<br>308 km - 17.01±4.31<br><br>Creatinine<br>Marathon - 1.06±0.10<br>100 km- 1.07±0.11<br>308 km - 1.03±0.05<br><br>S-CK<br>Marathon - 132.76±55.17<br>100 km- 108.58±40.76<br>308 km - 131.75±39.34	100 km- 32.31±4.87<br>308 km - 24.03±4.88<br><br>Creatinine<br>Marathon - 1.27±0.17<br>100 km- 1.27±0.24<br>308 km - 1.07±0.09<br><br>S-CK<br>Marathon - 274.17±70.95<br>100 km- 2983.17±5.56<br>308 km - 4970.31±2222.48	Change in SCr significantly higher after marathon and 100km ultra (medium to high intensity) compared to 308km	(20-23°C) and humidity (40-50%)			
Zyl-Smit 2000	Male 1<br>40 years old	Comrades Ultramarathon	NS	1= delayed presentation to medical staff – 9 days post-race<br><br>10 days post-race - SCr- 713 µmol/L, urea 32.7 mrnol/l, potassium<br>CK- NS<br>Urine- 2+ protein 1+ blood, moderate granular casts<br><br>Ultrasound = bilaterally unobstructed and moderately enlarged kidneys	Rhabdomyolysis (presumed)<br><br>acute tubular necrosis (ATN) and ARF	NS	treatment = Furosemide 250 mg intravenously followed by similar oral dose, hypertension - nifedipine 10 mg, 8-hourly<br><br>5 days post-presentation- plasma creatinine reduced - 260 µmol/l<br><br>After 10 days plasma	During race - 6 x paracetamol 500 mg/chlormezanone 100 mg (BessenoJ) tablets	1 x 2 mg loperarnide (Imodium) tablet before the race<br>Day 1 post race = 2 Bessenol tablets following 5 days - 11 Stopayne tablets (paracetamol / caffeine/ codeine phosphate / meprobamate)

							creatinine = 93 µmol/l & creatinine clearance = 102 ml/ min		
Jouffroy 2019	n = 47<br>43 ± 7 years	Ultra-distance race 80km	SCr - 90±14 µmol/L (km0)<br><br>SUrea - 4.7±4.5 mmol/L (km 0)	SCr increased to 136±32 µmol/ L (km 80—p<0.0001) = 52% increase<br>Mean SUrea increased to 22.8±12.0 mmol/L (km 80) (p<0.0001)<br><br>NGAL and Kidney injury molecule 1 (KIM-1) shown to progressively increase during race (p<0.05 vs baseline)<br>At day 9 NGAL and KIM1 urinary levels decreased but remained significantly higher than baseline levels	21km<br>2 (4%) = stage 1<br>0 = stages 2/3<br><br>53km<br>5 (11%) = stage 1<br>1 (2%) = stage 2<br>0 = stage 3<br><br>80km<br>3 (6%) = stage 1<br>0 = stages 2/3	+14˚C and clear weather	Day 9 – no significant difference from baseline	NS	NS
Lipman 2016	128 (28% women, mean age 39.6 ± 9 years)	4 Multistage ultramarathons	Baseline SCr - 0.72 (0.49 to 0.89) SD ±0.13 mg/dL	Stage 1 SCr - 1.20 (0.70 to 2.30) SD ± 0.28 mg/dL<br>Stage 3 SCr - 1.33 (0.70 to 2.90) SD ± 0.37mg/dL<br>Stage 5 SCr - 1.28 (0.70 to 2.70) SD ± 0.34 mg/dL<br>Mean SCr change at Stage 1 - 0.49 (0.02 to 1.54)<br>± 0.26<br>Stage 3 - 0.12 (20.50 to 1.50)<br>± 0.26	AKI status at Stages<br><br>Stage 1 – Normal = 37.5%<br>Risk AKI = 43.8%<br>AKI = 18.8%<br><br>Stage 3- Normal = 20.3%<br>Risk AKI = 47.7%<br>AKI = 32.0%	NS	NA	NS	NS

				Stage 5 - -0.05 (21.0 to 0.50)<br>± 0.24	Stage 5-<br>Normal = 23.4%<br>Risk AKI = 48.4%<br>AKI = 28.1%<br><br>per stage prevalence of AKI = 62.5% to 79.7%.<br><br>Slowest race finishers who were less likely to develop AKI, 82% less likely than those who finished in the top 10%.<br>Multivariate analysis found AKI was significantly				
Khodaee 2021	64, only 37 (both pre-and post-race results)	100km ultra race	mean (±SD) pre-race Cr – (n = 64) was 0.99 ± 0.15 mg/dL	Serum BUN and Cr increased (P < .001) post-race (n=37)	18 out of the 37 (49%) met the criteria for AKI.<br>Using fixed eGFR of 75 ml/ min per 1.73 m2 and an aged-based eGFR), resulted in 6 (16%) and 22 (59%) of 37		NA	Positive use of NSAIDS<br>NSAID 1-2 days prior to race =<br>18 AKI- n=3 yes<br>19 Not AKI – n=4 Yes<br><br>NSAIDS during race-<br>18 AKI- n=4 yes<br>19 Not AKI – n=4 Yes	

					runners = criteria for AKI, respectively<br><br>Different equations produced differing results with regard to the number of participants meeting criteria of AKI				
Lippi 2012	16	Ultramarathon	SCr (µmol/L) - 68 (58– 76)<br><br>eGFR, mL/min/1.73 m^2 - 112 (97 – 133)<br><br>Serum NGAL, ng/mL – 105 (86 – 158)<br><br>Urinary NGAL, ng/mL - 4.4 (0.5 – 33.9)	SCr (µmol/L)- 98 (76-118)<br>Mean Increase in SCr (µmol/L) – 30<br><br>eGFR, mL/min/1.73 m^2 - 75 (57 – 99)<br><br>Serum NGAL, ng/mL – 196 (139 – 290)<br><br>Urinary NGAL, ng/mL - 35.6 (12.5 – 86.3)	All post-race variables significantly different to baseline (p<0.001)<br><br>6 /16 athletes (38 %), = AKI	NS	NA	NS	NS
Noakes 1976	13<br>Group A – complete 160Km<br>Group B – completed 65 to 149 km	Ultramarathon	Group A<br>SCr- 1.1 (mg/100ml)<br>SUrea – 32.7 (mg/100ml)<br><br>Group B<br>SCr- 1.1 (mg/100ml)<br>SUrea – 30.2 (mg/100ml)	Group A<br>SCr- 1.5 (mg/100ml)<br>SUrea – 66.1 (mg/100ml)<br><br>Group B<br>SCr- 1.4 (mg/100ml)<br>SUrea – 52.7 (mg/100ml)	SUrea = doubled<br>SCr = increased by ~1/3	NS	NS	NS	NS

Irving 1989	2 males Experienced marathon runners	2-man 24 hr relay marathon	SCr (µmol/L) – 81 & 86	Post-race SCr (µmol/L) – 101 & 119<br>Repeated SCr (µmol/L) at 48hrs- both 92<br>Increase in SCr (µmol/L) – 20 & 23	SCr increased post-race<br><br>Cr clearance dropped	range of Wet Bulb Globe Temperature Index = 9.7-16.3ºC	Measures of renal dysfunction transient as normalised after 24hr	NS	NS
Irving 1990 - Transient oliguria with renal tubular dysfunction after a 90 km running race	5 (Group = 4 males, subject A = 1 female) Experienced marathon runners	Ultramarathon 90Km	Group<br>SCr (µmol/L) - 63.5 (±2.9)<br><br>Subject A<br>SCr (µmol/L) - 42 (±3)	Group<br>Race SCr (µmol/L)- 69.3 (±9.6)<br>Day 2 SCr (µmol/L) – 65.8 (±5.5)<br>Day 14 SCr (µmol/L)- 62.0 (±6.7)<br><br>Subject A<br>Race SCr (µmol/L)- 55.0<br>Day 2 SCr (µmol/L) – 53.0<br>Day 14 SCr (µmol/L)- 51.0	1= collapsed<br>Subject A = transient oliguria with renal tubular dysfunction and anuriadue to restricted fluid intake during race compared to rest of group = dehydration (-11% body weight) and urine creatinine excretion increase<br><br>Group = Urine flow rate, CrCl unchanged	Mean temperature – 17.9ºC (10.5 – 21.5 ºC)<br>Mean humidity – 60% (9 – 41%)	Subject A = Renal dysfunction measures persisted 14 days post-race although, full recovery 1 year later	NS	NS
Irving 1990 - Plasma volume and renal function during and after ultramarathon running	8 males experienced marathon runners	Ultramarathon	SCr (µmol/L) - 91	Post-race SCr (µmol/L)- 110<br>Repeated SCr (µmol/L) – at 24hrs = normal<br>Increase in SCr (µmol/L) – 19	SCr significantly increased on race day ($p<0.001$)	Mean temperature – 12.5ºC (7.3 – 18.3ºC)	Return to baseline within 24Hrs	NS	NS

Neumayr 2003	28 (amateur ultra-cyclists	Ultramaratho n cycle	SCr (µmol/L) - 88	Post-race SCr (µmol/L)- 106<br>Repeated SCr (µmol/L) at 24hrs- 106<br>Increase in SCr (µmol/L) – 18	SCr significantly elevated post-race, 20% (p<0.001)<br><br>Reduced renal perfusion mechanism responsible for renal impairment	temperatures ranged from 14–218C, and humidity from 55–85%	SCr remained elevated at 24hrs	NS	NS
Neumayr 2015	16 (males, world's best ultramarath on cyclists)	Ultramaratho n cycle	SCr (µmol/L) – 84 (±15)	Post-race SCr (µmol/L)- 111 (±19)<br>Repeated SCr (µmol/L) – 83 (±15)<br>Increase in SCr (µmol/L) – 26	Significant rise in SCr (+33%, p<0.001) post-race<br><br>Reduced renal perfusion mechanism responsible for renal impairment	temperatures ranged between -1 and 20°C	SCr Normalised within 24hrs	NS	NS
Christensen 2014	10	Ultramaratho n	SCr (µmol/L) – 60	Post-race SCr (µmol/L)- 80<br>Mean Increase in SCr (µmol/L) – 20	lack of an immediate post-exercise increase, although almost significant (P = 0.075)	NS	25% increase in SCr at 6hours post-race Normalisation at 48hrs post-race	NS	NS
Chilbkova 2015	113 ultra-runners and ultra-mountain bikers	24hr and 100km ultra-running and 24 hr and multistage ultra-	Male multistage bikers Plasma creatinine = 0.9 ± 0.1 (mg/dL) Plasma creatinine clearance =	Male multistage bikers Plasma creatinine = 1.2 ± 0.5 (mg/dL), +0.3 ± 0.5 (mg/dL), +33.3 ± 64.8%, p=0.01<br>Plasma creatinine clearance = 8953.6 ± 2447.9 (ml/min),	13 (11.5%) – EAH<br><br>6 (5%) – ER<br><br>2 (1.8%) – EAH & ER	NS	2 cases of elevated creatinine = No renal failure or medical intervention	NS	NS

		mountain bike races	10849.4 ± 1502.9<br>1009.4 (ml/min)<br><br>Male 24hrs bikers<br>Plasma creatinine = 0.9 ± 0.1<br>207.5 (mg/dL)<br>Plasma creatinine clearance = 10657.2 ± 1614.2 (ml/min)<br><br>Male 24hrs runners<br>Plasma creatinine = 0.9 ± 0.1(mg/dL)<br>Plasma creatinine clearance = 10657.2 ± 1614.2(ml/min)<br><br>Male 100km runners<br>Plasma creatinine = 0.9 ± 0.2(mg/dL)<br>Plasma creatinine clearance = 8985.3 ± 2219.0(ml/min)	−1895.8 ± 2158.7 (ml/min), −17.4 ± 19.3%, p<0.01<br><br>Male 24hrs bikers<br>Plasma creatinine = 1.2 ± 0.2 (mg/dL), +0.3 ± 0.2(mg/dL), +32.7 ± 21.8%, p<0.01<br>Plasma creatinine clearance = 9623.2 ± 1837.4(ml/min), −1034.1 ± 1121.0(ml/min), −9.8 ± 11.7%, p<0.05<br><br>Male 24hrs runners<br>Plasma creatinine = 0.9 ± 0.2 (mg/dL), +0.0 ± 0.1(mg/dL), +10.6 ± 12.7%, p=0.06<br>Plasma creatinine clearance = 9623.2 ± 1837.4(ml/min), −1034.1 ± 1121.0(ml/min), −9.8 ± 11.7%, p<0.05<br><br>Male 100km runners<br>Plasma creatinine = 1.1 ± 0.3<br>4357.1(mg/dL), +0.2 ± 0.3(mg/dL), + 25.8 ± 48.1%, p=0.05<br>Plasma creatinine clearance = 7327.7 ± 2080.2(ml/min), −1657.5 ± 2146.8(ml/min), −16.7 ± 17.9%, p<0.05	All cases of EAH and ER = ultra-runners<br><br>2 cases of elevated creatine >normal (~2.0 mg/dL or x1.5 estimated baseline)				

Lecina 2021	1 male (42yrs)	786 km multi-stage ultra-trail race	SCr = 0.88 (mg/dL)<br><br>Creatine kinase = 94 UI/L	SCr = 1.13mg/dL (+28.41%)<br><br>Creatine kinase = 1099 UI/L (+1069.15%)	ER<br><br>No AKI	Temperature 13.08 – 17.69 ºC<br>humidity 60.16–70.87%	Post-race day 2<br>SCr = 0.98mg/dL (+11.36%)<br>Creatine kinase = 478 UI/L (+408.51%)<br><br>Day 9<br>SCR = 0.84mg/dL (−4.55%)<br>Creatine kinase = 109UI/L (+15.96%)	NS	NS

AKI: acute kidney injury, ER: exertional rhabdomyolysis, EAH: exercise-associated hyponatraemia, NSAIDs: non-steroidal anti-inflammatory drugs, GFR: glomerular filtration rate, SCr: serum creatinine, S-CK: creatinine kinase, BUN: blood urea nitrogen, NGAL: neutrophil gelatinase-associated lipocalin, NS: not specified, Δ: change in
